# Evaluating the hypertension care cascade in middle-aged and older adults in The Gambia: findings from a nationwide survey

**DOI:** 10.1016/j.eclinm.2023.102226

**Published:** 2023-09-20

**Authors:** Modou Jobe, Islay Mactaggart, Abba Hydara, Min J. Kim, Suzannah Bell, Omar Badjie, Mustapha Bittaye, Pablo Perel, Andrew M. Prentice, Matthew J. Burton

**Affiliations:** a Medical Research Council Unit The Gambia at London School of Hygiene and Tropical Medicine, Fajara, The Gambia; b International Centre for Eye Health, London School of Hygiene & Tropical Medicine, UK; c Sheikh Zayed Regional Eye Care Centre, Kanifing, The Gambia; d International Statistics and Epidemiology Group, Department of Infectious Disease Epidemiology, London School of Hygiene and Tropical Medicine, London, UK; e Moorfields Eye Hospital NHS Foundation Trust, London, UK; f Directorate of Health Promotion & Education, Ministry of Health, The Gambia; g Directorate of Health Services, Ministry of Health, The Gambia; h Department of Obstetrics and Gynaecology, Edward Francis Small Teaching Hospital, Banjul, The Gambia; i Department of Non-communicable Disease Epidemiology, London School of Hygiene & Tropical Medicine, UK; j National Institute for Health Research Biomedical Research Centre for Ophthalmology at Moorfields Eye Hospital NHS Foundation Trust and UCL Institute of Ophthalmology, London, UK

**Keywords:** Hypertension, Cascade of care, Epidemiology, Prevalence, Awareness, Treatment, Control, The Gambia

## Abstract

**Background:**

Hypertension is a major public health problem in sub-Saharan Africa with poor treatment coverage and high case-fatality rates. This requires assessment of healthcare performance to identify areas where intervention is most needed. To identify areas where health resources should be most efficiently targeted, we assessed the hypertension care cascade i.e., loss and retention across the various stages of care, in Gambian adults aged 35 years and above.

**Methods:**

This study was embedded within the nationally representative 2019 Gambia National Eye Health Survey of adults ≥35 years. We constructed a hypertension care cascade with four categories: prevalence of hypertension (defined as systolic blood pressure ≥140 mmHg and/or diastolic blood pressure ≥90 mmHg, and/or current use of medication prescribed for hypertension); those aware of their diagnosis; those treated; and those with a controlled blood pressure (defined as blood pressure <140/90 mmHg). Analyses were age- and sex-standardised to the population structure of The Gambia. Logistic regression was used to assess the socio-demographic factors associated with prevalence, awareness, treatment and control of hypertension.

**Findings:**

Of 9171 participants with data for blood pressure, the prevalence of hypertension was 47.0%. Among people with hypertension, the prevalence of awareness was 54.7%, the prevalence of hypertension treatment was 32.5%, and prevalence of control was 10.0% with little difference between urban and rural residence. The cascade of care performance was better in women. However, there was no difference in achieving blood pressure control between men and women who were receiving treatment. Female sex, older age and higher body mass index were associated with higher hypertension awareness whilst having an occupation compared to being unemployed was associated with higher odds of being treated. Patients in the underweight category had higher odds of achieving blood pressure control.

**Interpretation:**

There is a high prevalence of hypertension and low performance of the health care system that impact on the hypertension care cascade among middle-aged and older adults in The Gambia. Addressing the full cascade will be paramount especially in reducing the mounting prevalence and improving diagnosis of patients with hypertension, where the greatest dividends will be gained.

**Funding:**

The 10.13039/100017151Queen Elizabeth Diamond Jubilee Trust, 10.13039/100010269Wellcome Trust.

**Research in context**

**Evidence before this study**

We searched PubMed for articles using a combination of search terms “hypertension”, “epidemiological studies”, “cascade of care” and “Africa South of the Sahara” on 10th February 2023 with no language or date restriction. Despite the availability of studies on prevalence and determinants of hypertension in sub-Saharan Africa, there are only a few on the hypertension care cascade in middle aged and older adults who are disproportionately affected.

**Added value of this study**

Our study provides a comprehensive population-based assessment of the hypertension care cascade and associated factors in middle-aged and older adults in The Gambia for the first time. This study highlights a remarkably high prevalence of hypertension and a poor cascade of care performance in The Gambia.

**Implications of all the available evidence**

The findings of this large nationally representative study suggest an urgent need for a comprehensive hypertension strategy, including but not limited to increased public awareness campaign on hypertension, and adoption of healthy dietary and lifestyle strategies, complemented by meaningful government policies, training and recruitment of more healthcare personnel, as well as adopting task-shifting approaches to increase access to management of hypertension.

## Introduction

Hypertension affects over 1.39 billion people globally, more than 75% of whom (1.04 billion people) live in low- and middle-income countries (LMICs).^1^^,^^2^ Sub-Saharan Africa (SSA) is disproportionately affected compared to other LMICs.^3^ The World Health Organisation estimates that 46% of the population aged 25 years and above in SSA have hypertension.^4^ In SSA as elsewhere, hypertension poses significant direct and indirect economic costs to individual patients, their families and to national economies.^5^, ^6^, ^7^

The cascade of care framework is a metric used for assessing the retention and loss of patients respectively across the stages of care necessary for achieving a treatment outcome. It is widely used to identify and quantify care gaps in chronic infectious and non-communicable diseases such as HIV/AIDS,^8^ tuberculosis,^9^ hepatitis C^10^ and diabetes.^11^ The cascade of care has also been used to evaluate hypertension care and has been found to be poor across all LMIC settings. Data reported from 1.1 million participants living in 44 LMICs show an overall hypertension prevalence of 17.5%, among whom 39.2% were aware of their diagnosis, 29.9% had received treatment, and only 10.3% of these had their blood pressure adequately controlled.^5^ In SSA however, control rates were less than 5% of patients in nearly two-thirds of countries^5^ and was reported to be 4% in The Gambia according to data from the 2010 WHO STEPwise approach to NCD risk factor surveillance (STEPS survey).^12^ According to Mills et al., high-income countries have approximately double the awareness (67.0% vs 37.9%) and treatment (55.6% vs 29.0%) rates and four times the rates of adequate blood pressure control among people with hypertension (28.4% vs 7.7%) compared to LMICs.^13^ This therefore calls for action by all stakeholders to contribute to improving detection, diagnosis, management and control especially in SSA.^14^

Despite multiple studies on the prevalence and determinants of hypertension in The Gambia, there is no information on awareness, treatment and control of hypertension to the best of our knowledge. The Gambia recently launched a 5-year multi-sectoral strategic plan to reduce, among others, cardiovascular and other NCDs by one-third by 2027.^15^ To achieve these objectives, there is a need for up-to-date evidence on hypertension care gaps to identify areas needing intervention, to assess performance level and formulate strategies for improvements. We conducted the present study in adults aged ≥35 years to determine the gaps in the hypertension care cascade and their associated factors in The Gambia.

## Methods

The present analysis was part of a NCD survey embedded into the 2019 Gambia National Eye Health Survey. The detailed study protocol is reported elsewhere.^16^ A multistage sampling strategy based on the 2013 Gambia Population and Housing Census data was used to identify a nationally representative sample of adults aged ≥35 years. The census enumeration areas were used as clusters, stratified into urban and rural. The clusters were selected to reflect the regional population using probability proportionate to size sampling methods. The selected clusters were segmented into groups of 30 participants. One group was subsequently selected at random. Detailed study information was provided to selected participants prior to obtaining a signed or thumb printed informed consent. They were subsequently invited to a central location on a given day for data collection.

The study protocol was approved by the Joint MRC/Gambia Government Ethics Committee (SCC 1635) and the London School of Hygiene & Tropical Medicine Ethics Committee (Ref 16172).

### Data collection procedures

Data were collected by trained study staff using a pre-tested questionnaire and captured electronically using the Open Data Kit (ODK) application installed on Android tablets. We collected socio-demographic (age, sex, highest level of education attained, ethnic group, marital status, occupation) and economic information from participants. We also collected data on cardiovascular risk factors such as smoking, alcohol consumption, history of hypertension and diabetes and current medication use for hypertension and diabetes.

We measured height to the nearest 0.1 cm with the participant standing fully erect against a portable stadiometer (Leicester Height Measure, Seca, Hamburg, Germany) and without footwear or headwear. Weight was measured to the nearest 0.01 kg using portable scales (Seca, Hamburg, Germany). Blood pressure was measured with the participant seated after resting for at least 10 min, with their arm supported at the level of the heart and resting on a surface. Measurement was taken in triplicate using automated OMRON-Healthcare 10 Series blood pressure monitors. The blood pressure measurements were taken 5 min apart, and the average of the last two measurements was used for analysis.

### Explanatory variables

Males and females were categorised into 6 age bands. Level of education was defined according to the highest level attained in either conventional school or the madrassa system, pre-coded as: pre-school, madrassa (pre-school), primary (lower basic), madrassa (lower basic), secondary (upper basic, junior, senior), secondary (madrassa), higher (tertiary, university, college), vocational, non-standard curriculum. These were further categorised into pre-school/no school, primary, secondary/vocational, higher, don't know/other, and non-formal/Quranic. Ethnicity was categorised based on which Gambian ethnic group participants identify themselves with. We recorded marital status as never married, currently married, widowed or divorced. Data on occupation was obtained in pre-coded categories as: professional/technical/managerial, clerical, sales and services, skilled manual, unskilled manual, domestic service, agriculture, and other. We further categorised this as Unemployed, Manual, Trades, Professional, Other and Retired/Old age. We used socio-economic data to calculate wealth quintiles using the EquityTool as previously described.^16^^,^^17^ Alcohol use was defined as any self-report of alcohol consumption in the past 12 months. Smoking status was categorised, as self-reported by participants as never smoker, current smoker and past smoker. Body mass index (BMI) was calculated as weight in kilograms divided by height in metres squared. Based on BMI, participants were categorised as underweight (<18 kg/m^2^), normal weight (18–24.9 kg/m^2^), overweight (25–29.9 kg/m^2^), and obese (≥30 kg/m^2^).

### Outcome variables

Hypertension was defined as systolic blood pressure of ≥140 mmHg and/or diastolic blood pressure ≥90 mmHg, ever diagnosed of hypertension, and/or current use of medication prescribed for hypertension. Participants were classified as aware if they reported having been diagnosed with hypertension by a health professional. Patients were regarded as treated if they reported currently receiving medication for hypertension. Patients with a systolic blood pressure of <140 mmHg and <90 mmHg were considered as having a controlled blood pressure and those not meeting these criteria as uncontrolled. We calculated this separately for the overall sample with hypertension regardless of treatment status for hypertension, and amongst those receiving treatment.

### Statistical analysis

This was a nationwide eye health and comorbidities survey where the sample size was calculated to detect disease prevalence as low as 0.5% with a power of 80% and a confidence interval of 95%. Further information on sample size calculation is detailed elsewhere.^16^

We accounted for the multistage design and conducted weighting to account for age, sex, and the clusters of the sample. Poststratification sample weights were calculated to account for the disproportionate age-sex sampling by 5-year band. Sample weights were created to generalize the findings to the 2013 Gambia Census. All weights were then multiplied with the cluster selection probabilities. During data collection, we addressed the potential bias with missing data by re-approaching non-respondents in clusters that had more than 50% missing data. As a result, all clusters in our survey had a higher than 50% response rate. For the remaining missing data in clusters that had more than 30 participants and less than 50% missing data, we conducted imputation with the most frequently observed value in the same cluster. We confirmed that prevalence of vision impairment by wealth quintile remained similar before and after imputation. More information on weighting and the approach to handling missing data is reported elsewhere.^16^ We estimated the prevalence of hypertension from the overall population, the prevalence of awareness among those with hypertension, the prevalence of treatment among those who were aware of their condition and the prevalence of control both among those on treatment and among all those with hypertension. Logistic regression was used to assess the factors associated with each outcome above. We then estimated the overall care cascade, keeping the denominator constant (population with hypertension) throughout in assessing the cumulative losses at each step of the cascade. We included all explanatory variables, determined *a priori* as potential confounders, in a multivariate model. All analyses were conducted in Stata software version 17 (Stata Corporation, College Station, TX, USA).

### Role of the funding source

The funders of the study had no role in study design, data collection, analysis, data interpretation, writing the report or the decision to submit for publication.

## Results

### Population characteristics

We enumerated a total of 11,127 in this nationwide survey of whom 9788 (88%) took part. In the present analysis, we excluded 600 (6.1%) participants with either missing household data or incomplete data and a further 17 (0.2%) participants with missing hypertension data. The present analysis therefore included 9171 participants who had data on hypertension status. Table 1 summarises the socio-demographic characteristics of participants with hypertension. Characteristics of the participants without hypertension are summarised in Supplementary Table S1. Urban men with hypertension were more likely to have attained secondary or higher level of education than others. Women were more likely to have lost their spouse than men (30.9% vs 0.9%) and more men were in a marital relationship (95.2% vs 66.6%). Men were more likely to be involved in professional jobs. Compared to their urban counterparts, rural participants were of lower socio-economic status, with none being found in the richest quintile. Obesity was present in 645 (15.7%) patients; predominantly in women in whom it was five-fold more common than in men (25.5% vs 5.1%). This difference was more pronounced in urban than in rural areas. Alcohol consumption was generally low with only 1.3% reporting consumption during the past 12 months. Overall, 7% of hypertensive patients were current smokers. This was almost exclusively in men compared to women (14.6% vs 0.1%), a difference that persisted when divided into urban (15.5% vs 0%) and rural (13.5% vs 0.2%) settlements. Similar patterns were also observed among previous smokers.

**Table 1:** Age and sex-standardised socio-demographic characteristics of participants with hypertension weighted for cluster size.

	Total			Urban (N = 2382)		Rural (N = 2031)	
	All (N = 4413)	M (N = 2100)	W (N = 2313)	M (N = 1034)	W (N = 1348)	M (N = 1065)	W (N = 966)
**Age (years)**							
Mean (SE)	54.3 (0.3)	54.6 (0.4)	54.0 (0.3)	55.1 (0.6)	53.9 (0.4)	54.2 (0.5)	54.1 (0.5)
35–44	1246 (28.2%)	570 (27.1%)	676 (29.3%)	274 (26.5%)	382 (28.4%)	295 (27.7%)	294 (30.5%)
45–54	1203 (27.3%)	553 (26.4%)	649 (28.1%)	247 (23.9%)	399 (29.6%)	305 (28.7%)	251 (26.0%)
55–64	894 (20.3%)	472 (22.5%)	422 (18.3%)	260 (25.2%)	261 (19.4%)	212 (19.9%)	162 (16.8%)
65–74	619 (14.0%)	310 (14.7%)	309 (13.4%)	160 (15.5%)	159 (11.8%)	149 (14.0%)	149 (15.5%)
75–84	309 (7.0%)	140 (6.7%)	169 (7.3%)	65 (6.3%)	94 (7.0%)	75 (7.0%)	75 (7.8%)
85+	142 (3.2%)	56 (2.3%)	86 (3.7%)	27 (2.6%)	52 (3.9%)	29 (2.7%)	34 (3.6%)
**Level of education attained**							
Pre-school/no school	842 (19.1%)	331 (15.8%)	511 (22.1%)	161 (15.6%)	317 (23.5%)	169 (15.9%)	194 (20.1%)
Primary	386 (8.8%)	189 (9.0%)	197 (8.5%)	99 (9.6%)	152 (11.3%)	90 (8.4%)	46 (4.8%)
Secondary/vocational	594 (13.5%)	399 (19.0%)	195 (8.4%)	267 (25.9%)	161 (11.9%)	133 (12.5%)	35 (3.6%)
Higher	154 (3.5%)	125 (6.0%)	29 (1.3%)	107 (10.4%)	27 (2.0%)	19 (1.8%)	2 (0.3%)
Don't know/other	92 (2.1%)	28 (1.3%)	64 (2.8%)	5 (0.5%)	27 (2.0%)	23 (2.2%)	36 (3.4%)
Non-formal/Quranic	2346 (53.2%)	1029 (49.0%)	1317 (56.9%)	394 (38.1%)	665 (49.3%)	632 (59.3%)	652 (67.4%)
**Ethnicity**							
Mandinka	1640 (37.2%)	720 (34.3%)	920 (39.8%)	406 (39.2%)	598 (44.4%)	314 (29.5%)	324 (33.5%)
Wollof	624 (14.1%)	313 (14.9%)	310 (13.4%)	112 (10.8%)	147 (10.9%)	200 (18.8%)	163 (16.9%)
Jola/Karoninka	469 (10.6%)	232 (11.0%)	237 (10.3%)	144 (14.0%)	158 (11.7%)	88 (8.2%)	80 (8.3%)
Fula/Tukulor/Lorobo	945 (21.4%)	508 (24.2%)	437 (18.9%)	219 (21.2%)	219 (16.3%)	288 (27.1%)	217 (22.5%)
Sarahuleh	404 (9.2%)	179 (8.5%)	225 (9.7%)	59 (5.7%)	90 (6.6%)	119 (11.2%)	135 (14.0%)
Others	331 (7.5%)	148 (7.0%)	183 (7.9%)	93 (9.0%)	137 (10.1%)	55 (5.2%)	47 (4.9%)
**Marital status**							
Never married	62 (1.4%)	51 (2.4%)	10 (0.4%)	40 (3.9%)	8 (0.6%)	11 (1.1%)	2 (0.2%)
Married/living together	3540 (80.2%)	2000 (95.2%)	1540 (66.6%)	959 (92.8%)	883 (65.5%)	1039 (97.6%)	658 (68.1%)
Widowed	733 (16.6%)	18 (0.9%)	715 (30.9%)	11 (1.15%)	415 (65.5%)	7 (0.7%)	301 (31.1%)
Divorced/separated	79 (1.8%)	31 (1.5%)	48 (2.1%)	24 (2.3%)	42 (3.1%)	7 (0.7%)	6 (0.6%)
**Occupation**							
Unemployed	726 (16.5%)	253 (12.0%)	473 (20.5%)	183 (11.7%)	327 (24.3%)	70 (6.6%)	147 (15.2%)
Manual	2136 (48.4%)	952 (45.3%)	1184 (51.2%)	210 (20.4%)	502 (37.2%)	736 (69.1%)	680 (70.4%)
Trades	1046 (23.7%)	570 (27.1%)	476 (20.6%)	419 (40.5%)	408 (30.3%)	153 (14.4%)	71 (7.4%)
Professional	248 (5.6%)	210 (10.0%)	38 (1.7%)	144 (14.0%)	32 (2.4%)	67 (6.3%)	6 (0.6%)
Other	66 (1.5%)	55 (2.6%)	12 (0.5%)	35 (3.4%)	9 (0.6%)	20 (1.9%)	3 (0.3%)
Retired/old age	190 (4.3%)	61 (2.9%)	129 (5.6%)	42 (4.1%)	70 (5.2%)	19 (1.8%)	59 (6.1%)
**Wealth quintile**							
1 (Lowest)	423 (9.6%)	216 (10.3%)	207 (9.0%)	20 (1.9%)	13 (0.9%)	194 (18.3%)	193 (20.0%)
2	648 (14.7%)	361 (17.2%)	287 (12.4%)	61 (5.9%)	56 (4.2%)	298 (28.0%)	229 (23.7%)
3	1087 (24.6%)	538 (25.6%)	549 (23.8%)	88 (8.5%)	105 (7.8%)	446 (41.9%)	441 (45.6%)
4	1035 (23.4%)	497 (23.7%)	537 (23.2%)	373 (36.1%)	436 (32.3%)	126 (11.8%)	104 (10.8%)
5 (Highest)	1220 (27.6%)	487 (23.3%)	732 (31.7%)	492 (47.6%)	739 (54.8%)	0	0
**BMI**							
Mean (SE)	24.9 (0.1)	23.3 (0.1)	26.4 (0.1)	23.9 (0.2)	27.7 (0.2)	27.7 (0.2)	24.6 (0.2)
Underweight	236 (5.7%)	138 (7.1%)	98 (4.5%)	41 (4.3%)	40 (3.2%)	96 (9.7%)	57 (6.4%)
Normal	2132 (51.8%)	1227 (62.7%)	906 (41.9%)	575 (60.0%)	430 (34.0%)	650 (65.2%)	474 (52.9%)
Overweight	1106 (26.9%)	492 (25.2%)	614 (28.4%)	288 (30.0%)	394 (31.1%)	205 (20.6%)	221 (24.7%)
Obese	645 (15.7%)	100 (5.1%)	545 (25.2%)	55 (5.7%)	403 (31.8%)	45 (4.6%)	144 (16.1%)
Missing^a^	297 (6.7%)	145 (6.9%)	152 (6.6%)	78 (7.5%)	84 (6.2%)	67 (6.3%)	69 (7.1%)
**Alcohol consumption in the past year**							
Never	4355 (98.7%)	2058 (98.1%)	2297 (99.3%)	1004 (97.2%)	1343 (99.6%)	1051 (98.8%)	956 (98.9%)
Ever	58 (1.3%)	43 (2.0%)	16 (0.7%)	29 (2.8%)	5 (0.4%)	12 (1.3%)	10 (1.1%)
**Smoking status**							
Current smoker	308 (7.0%)	306 (14.6%)	2 (0.1%)	160 (15.5%)	0 (0.0%)	146 (13.7%)	2 (0.2%)
Never smoked	3765 (85.3%)	1454 (69.3%)	2311 (99.95)	687 (66.5%)	1348 (100%)	765 (71.9%)	964 (99.8%)
Previous smoker	340 (7.7%)	340 (16.2%)	0 (0.0%)	186 (18.0%)	0 (0.0%)	153 (14.4%)	0 (0.0%)

Data are in n (%); Abbreviations: M = men; W = women; SE = standard error.

a Missing data are in n(%) of total participants, and are not included in calculation of prevalence estimates.

### Prevalence and socio-demographic predictors of hypertension

**Fig. 1:**
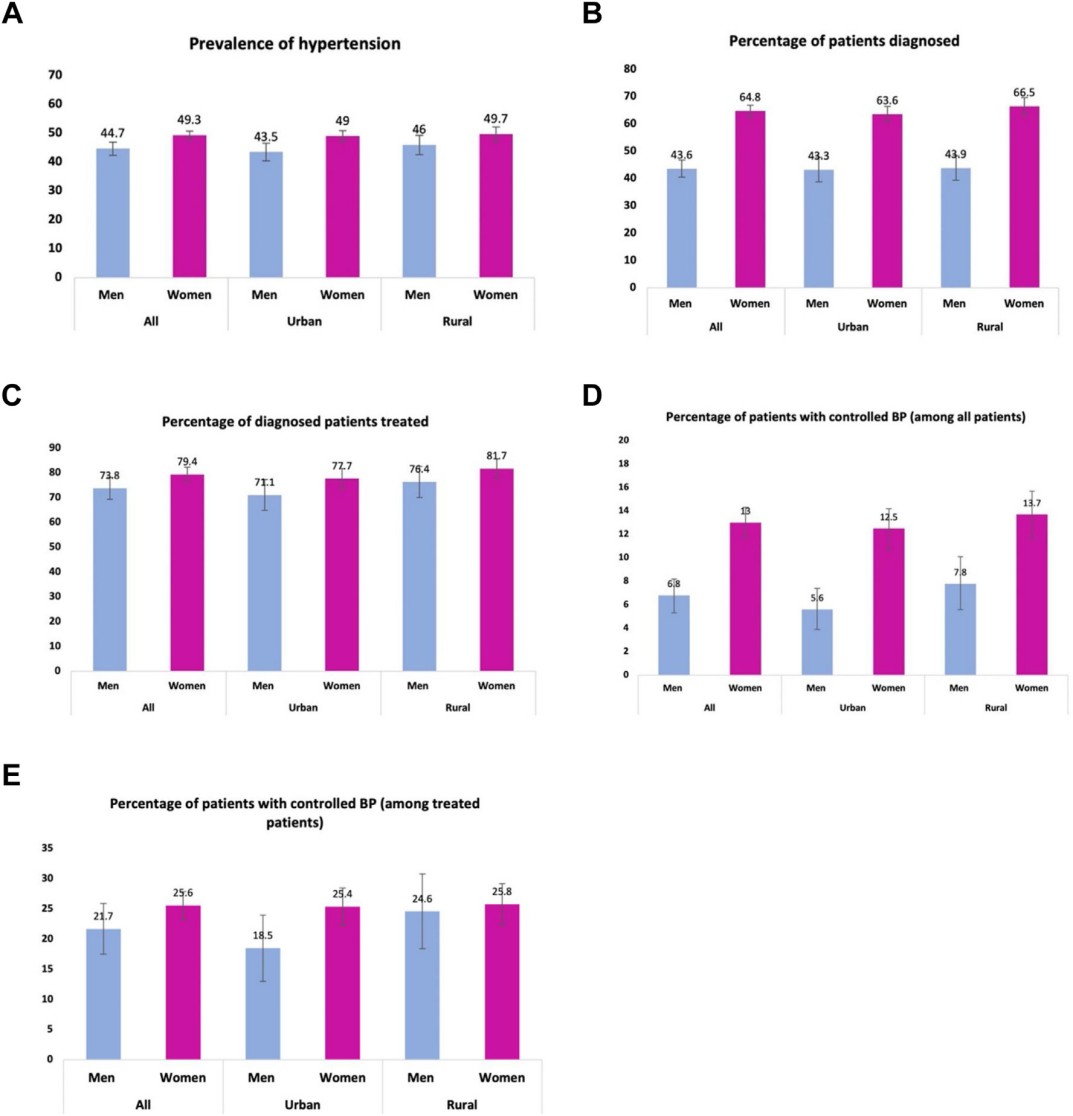
Prevalence, diagnosis, treatment, and control of hypertension by sex and location. Figure uses age and sex standardised rates accounting for multistage sampling design. y-axis: percentage of participants.

The overall prevalence of hypertension was 47.0% (95% confidence interval: 45.6–48.5) and was higher in women [49.3% (47.8–50.8)] compared with men [44.7% (42.4–47.0)] (Table 2, Fig. 1A, Supplementary Table S2).

**Table 2:** Age and sex-standardised prevalence, diagnosis, treatment, and control of hypertension by socio-demographic factors.

Variable	Number of participants	Number of individuals with hypertension	Prevalence of hypertension (%)	Proportion with diagnosed hypertension (aware) (%)	Proportion of diagnosed patients receiving treatment (%)	Controlled	
						Among all hypertensives (%)	Among treated (%)
**Overall**	9171	4313	47.0 (45.6–48.5)	54.7 (52.8–56.6)	70.4 (67.6–73.3)	10.0 (9.0–11.1)	24.2 (22.0–26.4)
**Sex**							
Men	4589	2052	44.7 (42.4–47.0)	43.6 (40.5–46.8)	69.5 (65.1–73.9)	6.8 (5.3–8.2)	21.7 (17.5–25.9)
Women	4582	2261	49.3 (47.8–50.8)	64.8 (62.7–66.9)	71.0 (67.9–74.0)	13.0 (11.7–14.3)	25.6 (23.3–27.9)
**Residence**							
Urban	4966	2308	46.5 (44.7–48.3)	54.8 (52.3–57.3)	68.8 (64.7–73.0)	9.5 (8.2–10.9)	23.2 (20.3–26.1)
Rural	4204	2005	47.7 (45.3–50.0)	54.7 (51.8–57.5)	72.4 (68.4–76.4)	10.6 (9.0–12.3)	25.3 (21.9–28.7)
**Age group**							
35–44	3992	1218	30.5 (28.7–32.4)	42.0 (38.3–45.7)	60.9 (55.9–65.9)	11.3 (9.1–13.4)	37.0 (31.5–42.5)
45–54	2454	1175	47.9 (45.3–50.5)	56.4 (52.7–60.0)	69.6 (64.8–74.4)	10.9 (8.9–12.9)	25.9 (21.3–30.4)
55–64	1349	874	64.8 (62.2–67.3)	59.3 (56.1–62.6)	73.9 (69.9–77.8)	9.6 (7.7–11.5)	20.8 (16.9–24.6)
65–74	808	605	74.9 (72.0–77.7)	63.8 (60.3–67.4)	75.4 (71.0–79.8)	8.6 (6.5–10.6)	17.4 (13.6–21.3)
75–84	391	302	77.3 (73.2–81.4)	64.7 (59.3–70.1)	81.1 (75.4–86.8)	7.4 (4.5–10.2)	13.9 (8.7–19.0)
85+	177	139	78.3 (70.9–85.8)	62.6 (51.7–73.6)	76.5 (64.2–88.8)	7.2 (1.8–12.5)	15.8 (4.4–27.1)
**Level of education attained**							
Pre-school/no school	1612	823	51.0 (47.8–54.3)	59.7 (55.7–63.6)	82.4 (78.7–86.1)	11.0 (8.9–13.2)	20.8 (16.8–24.8)
Primary	979	377	38.5 (34.5–42.5)	50.0 (43.9–56.2)	69.2 (62.0–76.3)	12.3 (8.6–16.1)	31.9 (23.5–40.3)
Secondary/vocational	1537	580	37.8 (34.5–41.0)	46.8 (41.7–52.0)	62.9 (55.9–70.0)	8.2 (5.8–10.7)	25.3 (18.3–32.4)
Higher	401	151	37.6 (31.0–44.1)	41.1 (31.3–50.8)	69.8 (55.4–84.2)	5.6 (0.9–10.3)	18.6 (4.3–32.9)
Don't know/other	155	89	57.9 (49.8–66.0)	58.5 (47.3–69.8)	78.0 (66.0–90.1)	14.5 (7.0–22.0)	34.8 (19.6–50.1)
Non-formal/Quranic	4488	2292	51.1 (49.0–53.1)	56.5 (54.0–59.0)	67.5 (63.8–71.3)	9.9 (8.5–11.3)	24.2 (21.3–27.1)
**Ethnicity**							
Mandinka	3413	1603	47.0 (44.9–49.0)	56.5 (53.7–59.4)	70.0 (66.0–74.0)	10.1 (8.4–11.7)	23.3 (19.9–26.6)
Wollof	1358	610	44.9 (41.4–48.4)	56.3 (51.5–61.0)	69.4 (63.6–75.2)	10.9 (7.8–14.0)	26.0 (19.8–32.1)
Jola/Karoninka	1026	459	44.7 (40.8–48.6)	48.8 (44.1–53.6)	65.9 (57.5–74.3)	9.1 (6.5–11.6)	26.0 (20.0–32.1)
Fula/Tukulor/Lorobo	2019	924	45.7 (42.5–49.0)	51.1 (47.0–55.3)	68.9 (63.4–74.5)	9.9 (7.6–12.1)	26.1 (21.1–31.2)
Sarahuleh	692	395	57.2 (52.5–61.8)	57.0 (50.4–63.5)	78.6 (72.8–84.3)	11.0 (8.1–13.8)	23.0 (16.8–29.3)
Others	664	323	48.7 (42.9–54.6)	58.9 (51.5–66.4)	74.7 (64.6–84.7)	8.8 (5.7–11.9)	20.1 (13.7–26.4)
**Marital status**							
Never married	208	60	28.9 (19.7–38.0)	22.5 (7.5–37.6)	65.9 (39.0–92.9)	4.5 (−4.1–12.1)	25.2 (−16.6–67.0)
Married/living together	7804	3459	44.3 (42.7–45.9)	52.5 (50.3–54.6)	68.7 (65.6–71.9)	9.9 (8.7–11.0)	25.2 (22.6–27.7)
Widowed	988	716	72.5 (69.9–75.2)	69.4 (66.1–72.8)	77.5 (73.4–81.6)	11.4 (9.0–13.7)	20.7 (16.7–24.7)
Divorced/separated	171	77	45.1 (37.2–52.9)	45.0 (32.8–57.2)	67.9 (53.8–82.1)	8.5 (11.2–15.8)	24.7 (5.9–43.6)
**Occupation**							
Unemployed	1049	710	67.6 (64.5–70.7)	64.4 (60.4–68.3)	67.4 (62.0–72.8)	9.9 (7.6–12.2)	21.8 (17.2–26.4)
Manual	4518	2088	46.2 (44.2–48.2)	54.6 (51.8–57.4)	72.3 (69.0–75.5)	11.2 (9.7–12.6)	26.1 (23.0–29.2)
Trades	2565	1022	39.8 (37.4–42.3)	49.6 (45.3–53.9)	63.6 (58.3–68.9)	9.1 (7.1–11.0)	26.1 (21.2–30.9)
Professional	646	243	37.6 (32.5–42.7)	40.9 (32.5–49.3)	71.6 (60.6–82.6)	8.9 (4.4–13.4)	27.8 (15.4–40.3)
Other	163	65	39.6 (26.4–52.9)	47.4 (32.0–62.8)	94.2 (86.4–1.02)	6.2 (−0.2–12.7)	13.9 (−0.5–28.3)
Retired/old age	229	186	81.3 (76.1–86.4)	68.5 (61.0–76.0)	87.6 (81.3–94.0)	6.0 (2.4–9.5)	10.0 (4.3–15.6)
**Wealth quintile**							
1 (Lowest)	862	413	47.9 (43.2–52.6)	52.9 (47.0–58.8)	67.6 (59.1–76.0)	11.1 (7.1–15.2)	28.8 (20.5–37.2)
2	1419	634	44.7 (41.4–47.9)	53.9 (48.6–59.3)	67.0 (60.3–73.8)	8.6 (5.7–11.5)	22.2 (15.1–29.2)
3	2238	1063	47.5 (44.6–50.4)	55.4 (51.6–59.1)	75.6 (71.5–79.7)	11.8 (9.7–13.9)	26.4 (22.3–30.5)
4	2140	1011	47.2 (44.5–50.0)	56.5 (52.3–60.6)	68.4 (63.0–73.9)	9.1 (7.2–11.1)	21.8 (17.4–26.1)
5 (Highest)	2511	1192	47.5 (45.2–49.8)	53.8 (50.5–57.0)	70.3 (65.4–75.3)	9.6 (7.8–11.5)	23.7 (19.8–27.5)
**Alcohol consumption in the past year**							
Never	9070	4256	46.9 (45.5–48.4)	54.9 (53.1–56.8)	70.3 (67.5–73.2)	10.1 (9.0–11.1)	24.2 (22.0–26.5)
Ever	101	57	56.3 (46.9–65.8)	38.8 (18.2–59.4)	83.4 (63.7–1.03)	6.0 (−0.5–12.5)	18.4 (3.8–33.0)
**Smoking status**							
Current smoker	894	301	33.7 (29.1–38.3)	31.5 (23.8–39.2)	69.9 (55.2–84.6)	6.7 (2.3–11.2)	29.2 (13.0–45.4)
Never smoked	7598	3680	48.4 (46.9–49.9)	57.3 (55.4–59.3)	70.0 (67.0–73.0)	10.4 (9.3–11.4)	23.9 (21.8–26.1)
Previous smoker	679	332	49.0 (43.1–54.9)	46.9 (38.4–55.3)	77.1 (68.4–85.7)	9.5 (5.4–13.5)	24.4 (14.0–34.7)
**BMI**							
Underweight	623	230	36.9 (32.0–41.8)	52.4 (44.4–60.3)	70.6 (61.1–80.1)	12.9 (7.6–18.3)	32.7 (21.0–44.4)
Normal	4893	2079	42.5 (40.6–44.4)	46.8 (44.2–49.3)	69.1 (65.3–72.8)	8.2 (7.0–9.5)	23.6 (20.4–26.8)
Overweight	2143	1079	50.3 (47.6–53.0)	58.3 (54.7–62.0)	71.8 (67.7–75.9)	10.1 (8.1–12.0)	22.4 (18.3–26.4)
Obese	1047	629	60.0 (57.2–62.9)	71.3 (67.8–74.8)	69.3 (64.6–74.0)	15.6 (12.9–18.4)	28.5 (24.0–32.9)

In multivariate analysis residence, age, ethnicity, marital status, and BMI were significantly associated with the odds of hypertension. There were higher odds of hypertension in rural [1.24 (1.02–1.52)] compared to urban areas. Compared with the youngest age group (35–44 years), there was steep rise in the odds of hypertension increasing to more than five-fold in people above 65 years with little further rise (Table 3). The Sarahuleh ethnic group had significantly higher odds of hypertension compared with the others [OR = 1.59 (1.26–2.00)]. Compared with the never married, hypertension was similar among the married/living together and divorced/separated patients but was significantly higher among the widowed. Compared to those in the normal BMI category, there was a lower odds of hypertension in the underweight [0.65 (0.51–0.82)] but higher odds in the overweight [1.54 (1.34–1.76)] and obese [2.46 (2.13–2.84)] (Table 3).

**Table 3:** Mutually adjusted analysis of factors associated with prevalence, diagnosis, treatment, and control of hypertension by socio-demographic factors.

Variable	Prevalence of hypertension	Proportion with diagnosed hypertension	Proportion of hypertensive patients treated	Controlled	
				Among all hypertensives	Among treated
**Sex**					
Men	1	1	1	1	1
Women	0.88 (0.76–1.03)	2.15 (1.75–2.64)	1.85 (1.27–2.66)	2.22 (1.57–3.15)	1.21 (0.81–1.81)
**Residence**					
Urban	1	1	1	1	1
Rural	1.24 (1.02–1.52)	1.15 (0.88–1.51)	1.25 (0.76–2.06)	1.06 (0.76–1.47)	0.99 (0.69–1.41)
**Age group (years)**					
35–44	1	1	1	1	1
45–54	1.99 (1.75–2.27)	1.91 (1.54–2.36)	1.26 (0.88–1.79)	0.96 (0.71–1.30)	0.59 (0.42–0.83)
55–64	3.63 (3.12–4.23)	2.28 (1.81–2.87)	1.62 (1.10–2.39)	0.90 (0.64–1.27)	0.45 (0.31–0.65)
65–74	5.22 (4.32–6.31)	2.59 (1.97–3.41)	1.56 (0.99–2.39)	0.81 (0.56–1.18)	0.37 (0.23–0.57)
75–84	5.23 (3.94–6.95)	2.65 (1.85–3.80)	2.43 (1.34–4.39)	0.72 (0.40–1.30)	0.28 (0.15–0.53)
85+	5.57 (3.14–9.86)	1.95 (1.04–3.64)	2.99 (0.96–9.30)	0.57 (0.18–1.78)	0.25 (0.08–0.84)
**Level of education attained**					
Pre-school/no school	1	1	1	1	1
Primary	0.91 (0.72–1.15)	0.89 (0.65–1.21)	0.49 (0.30–0.82)	1.05 (0.69–1.59)	1.28 (0.82–1.99)
Secondary/vocational	0.91 (0.73–1.13)	0.94 (0.70–1.27)	0.35 (0.22–0.55)	0.75 (0.50–1.13)	1.04 (0.66–1.63)
Higher	0.79 (0.55–1.13)	0.91 (0.52–1.58)	0.41 (0.17–1.02)	0.50 (0.19–1.38)	0.52 (0.17–1.57)
Don't know/other	1.24 (0.78–1.95)	0.82 (0.52–1.29)	0.54 (0.22–1.29)	1.10 (0.56–2.15)	1.43 (0.68–3.02)
Non-formal/Quranic	1.01 (0.85–1.19)	0.89 (0.71–1.11)	0.33 (0.22–0.48)	0.82 (0.62–1.10)	1.17 (0.86–1.59)
**Ethnicity**					
Mandinka	1	1	1	1	1
Wollof	0.95 (0.80–1.14)	1.07 (0.84–1.37)	1.02 (0.69–1.52)	1.23 (0.87–1.74)	1.32 (0.94–1.85)
Jola/Karoninka	0.84 (0.69–1.01)	0.74 (0.57–0.94)	0.35 (0.22–0.55)	0.85 (0.61–1.18)	1.10 (0.78–1.55)
Fula/Tukulor/Lorobo	1.07 (0.91–1.26)	0.90 (0.72–1.13)	0.41 (0.17–1.02)	1.02 (0.73–1.42)	1.17 (0.83–1.65)
Sarahuleh	1.59 (1.26–2.00)	0.96 (0.70–1.32)	0.54 (0.22–1.29)	0.96 (0.68–1.37)	0.91 (0.60–1.37)
Others	1.01 (0.75–1.35)	0.99 (0.70–1.40)	0.33 (0.22–0.48)	0.85 (0.54–1.35)	0.81 (0.48–1.38)
**Marital status**					
Never married	1	1	1	1	1
Married/living together	1.19 (0.73–1.94)	1.72 (0.64–4.64)	0.42 (0.07–2.63)	1.57 (0.19–12.77)	1.18 (0.79–17.68)
Widowed	1.77 (1.05–2.99)	1.59 (0.58–4.34)	0.48 (0.08–3.01)	1.71 (0.21–14.03)	1.37 (0.91–21.05)
Divorced/separated	1.34. (0.72–2.48)	1.23 (0.40–3.78)	0.41 (0.56–3.04)	1.30 (0.13–13.37)	1.33 (0.07–23.66)
**Occupation**					
Unemployed	1	1	1	1	1
Manual	0.72 (0.60–0.87)	0.82 (0.64–1.04)	1.51 (1.06–2.16)	0.98 (0.68–1.40)	0.95 (0.65–1.40)
Trades	0.65 (0.53–0.79)	0.81 (0.61–1.06)	1.09 (0.73–1.61)	0.83 (0.56–1.23)	0.85 (0.56–1.28)
Professional	0.73 (0.54–1.00)	0.84 (0.53–1.33)	2.17 (1.03–4.57)	1.33 (0.63–2.81)	1.22 (0.53–2.78)
Other	0.59 (0.32–1.07)	1.12 (0.54–2.32)	7.75 (1.53–39.30)	0.67 (0.17–2.65)	0.33 (0.07–1.55)
Retired/old age	1.18 (0.78–1.77)	0.92 (0.59–1.44)	2.07 (0.84–5.09)	0.51 (0.25–1.06)	0.51 (0.25–1.07)
**Wealth quintile**					
1 (lowest)	1	1	1	1	1
2	0.93 (0.73–1.18)	1.23 (0.87–1.73)	1.07 (0.62–1.86)	0.83 (0.51–1.33)	0.61 (0.40–0.99)
3	0.93 (0.73–1.17)	1.06 (0.78–1.44)	1.82 (1.08–3.09)	1.14 (0.74–1.76)	0.93 (0.60–1.42)
4	1.01 (0.77–1.32)	1.19 (0.83–1.72)	1.35 (0.73–2.52)	0.87 (0.52–1.46)	0.72 (0.43–1.20)
5 (richest)	1.05 (0.79–1.39)	0.89 (0.61–1.31)	1.24 (0.65–2.38)	0.85 (0.50–1.45)	0.88 (0.52–1.49)
**Alcohol consumption in the past year**					
Never	1	1	1	1	1
Ever	1.38 (0.83–2.30)	0.76 (0.37–1.55)	2.19 (0.39–12.30)	0.98 (0.31–3.11)	1.08 (0.36–3.27)
**Smoking status**					
Current smoker	0.80 (0.63–1.02)	0.71 (0.47–1.07)	1.26 (0.55–2.91)	1.29 (0.58–2.84)	1.73 (0.77–3.89)
Never smoked	1	1	1	1	1
Previous smoker	1.07 (0.81–1.42)	1.05 (0.69–1.58)	1.86 (1.01–3.43)	1.75 (0.99–3.08)	1.52 (0.77–2.98)
**BMI**					
Underweight	0.65 (0.51–0.82)	1.17 (0.84–1.64)	1.24 (0.68–2.26)	1.82 (1.13–2.93)	1.81 (1.06–3.07)
Normal	1	1	1	1	1
Overweight	1.54 (1.34–1.76)	1.63 (1.37–1.95)	1.20 (0.91–1.58)	1.17 (0.89–1.53)	0.86 (0.65–1.14)
Obese	2.46 (2.13–2.84)	2.48 (2.01–3.08)	1.11 (0.81–1.51)	1.69 (1.27–2.26)	1.14 (0.83–1.55)

### Prevalence and socio-demographic predictors of patients’ awareness of their hypertension

Among those with hypertension, 54.7% (52.8–56.6) were aware of their hypertension status with significantly higher awareness among women [64.8% (62.7–66.9)] compared to men [43.6% (40.5–46.8)] (Table 2 and Fig. 1B).

**Supplementary Figure S1:**
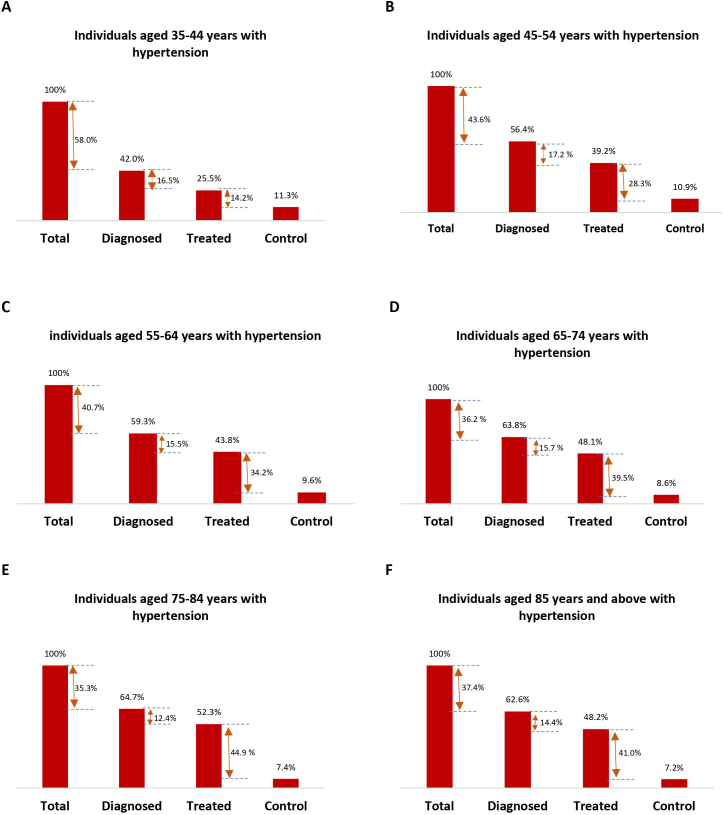

Sex, age and body mass index were associated with patients’ awareness of their diagnosis (Table 3). Awareness was significantly higher in women [OR 2.15 (1.75–2.64)] compared to men. We observed that those in the older age categories were more likely to be aware of their diagnosis compared to the youngest age category (35–44 years) (Supplementary Figure S1). The odds of awareness in overweight [1.63 (1.37–1.95)] and obese patients [2.48 (2.01–3.08)] was higher than in normal and underweight individuals (Table 3]. Those belonging to the Jola ethnic group were less likely to be aware of their diagnosis of hypertension compared to the Mandinka group (Table 3).

### Prevalence and socio-demographic predictors of treatment for hypertension

More than two-thirds (70.4% (67.6–73.3)) of those aware of their hypertension were receiving treatment.

Women were more likely to be receiving treatment [1.85 (1.27–2.66)] compared to men. Rural women were most likely to receive treatment whilst urban men were least likely to be on treatment (Fig. 1C, Supplementary Table S2). Compared to the unemployed, patients in manual, professional and other occupational categories were more likely to be treated for their hypertension.

### Prevalence and socio-demographic predictors of adequate BP control

Overall, only 10.0% (9.0–11.1) of people with hypertension had their blood pressure controlled but the rate was considerably higher among those receiving treatment [24.2% (22.0–26.4)].

Of those receiving treatment, those in the younger age categories were more likely to have a controlled blood pressure: 37.0% (31.5–42.5) in the youngest age group reducing to 15.8% (4.4–27.1) in those aged ≥85 years (Table 2 and Fig. 1D and E). The biggest loss in the cascade of care in those aged ≥55 years was between treatment to achieving controlled BP being 34.2%, 39.5%, 44.9% and 41.0% in the age groups 55–64, 65–74, 75–84 and ≥ 85 years respectively. This was 14.2% and 28.3% respective among those aged 35–44 and 45–54 tears respectively (Supplementary Figure S1). Women were more likely to achieve adequate BP control [OR = 2.22 (1.55–3.15)] compared to men, but there was no difference between sexes when restricted to those on treatment. There was no variation in BP control in the overall population by age group. When this was compared among those receiving treatment, those in the older age categories were much less likely to achieve BP control compared to the 35–44-year group with odds ratios between 0.37 and 0.25 for those over 65 years. There appears to be no effect of higher BMI on BP control among those receiving treatment compared with those in the normal category, but underweight patients were more likely to achieve BP control [1.81 (1.06–3.07)] (Table 3).

### Gaps in the hypertension care cascade

**Fig. 2:**
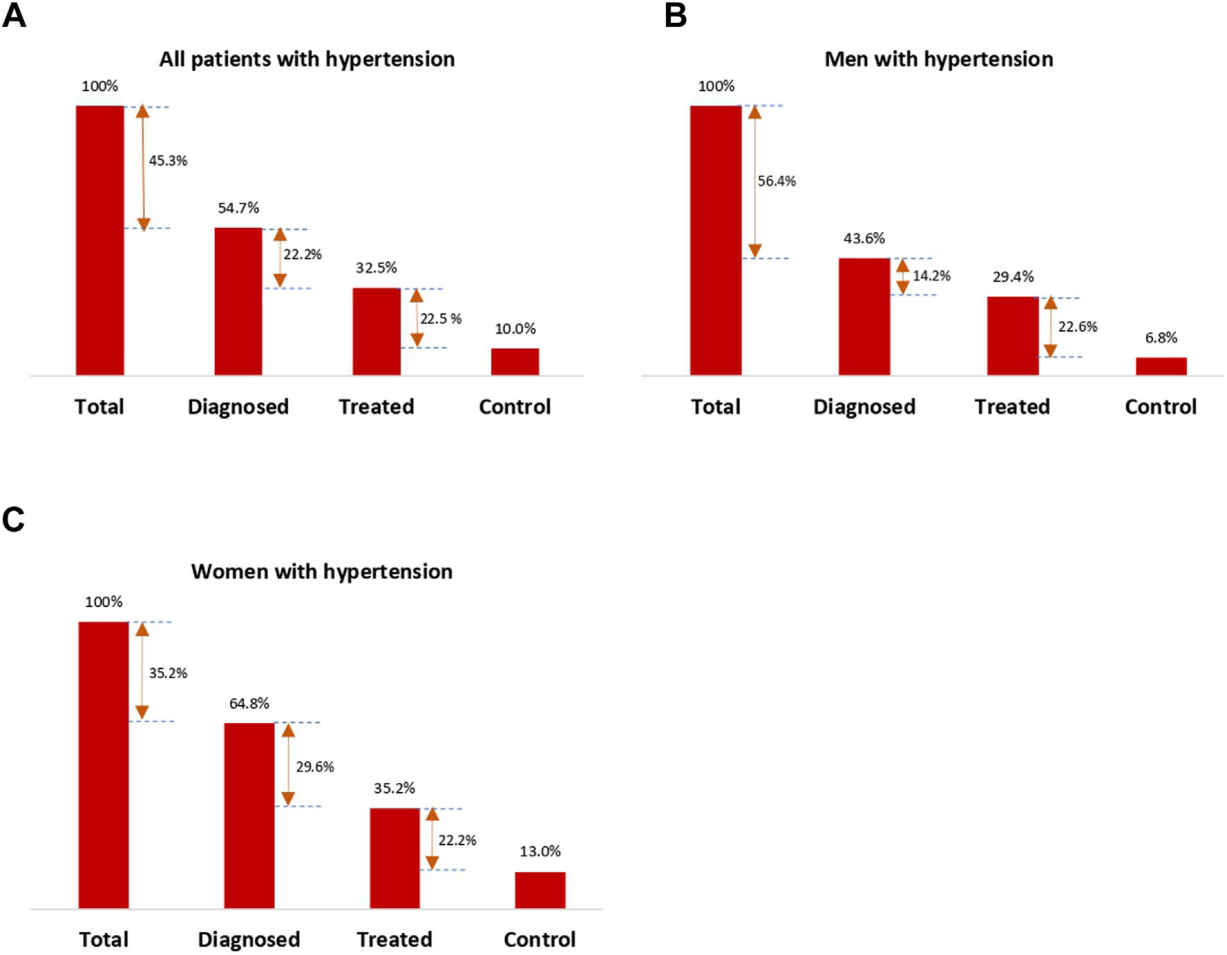
Hypertension care cascade in The Gambia of adults aged 35 years and above. The numbers between bars represent percentage loss at each step of the cascade. Total = total participants with hypertension.

Among hypertensive patients, the prevalence of awareness was 54.7%, the prevalence of hypertension treatment was 32.5% and prevalence of control was 10.0%. Among men with hypertension, 43.6% were aware of their diagnosis, 29.4% receiving treatment and 6.8% had their BP controlled. In women, 64.8% were aware of their hypertension, 35.2% were receiving treatment and 13.0% with a controlled BP (Table 2 and Fig. 2). Of the total 93.2% of men who were lost in the care cascade, 56.4% were at the diagnosis stage, 14.2% at the treatment stage and 22.6% at the control stage of the cascade respectively. Among the 87% of women not achieving adequate control the corresponding losses were 35.2%, 29.6% and 22.2% respectively (Fig. 2).

## Discussion

This nationally representative study found that nearly half of Gambian adults aged ≥35 years had hypertension. More than half of those were aware of their diagnosis of whom a little over two-thirds were receiving treatment for hypertension. However only 10% overall and nearly 25% of treated patients had a controlled blood pressure. This remarkably high prevalence and poor cascade of care performance calls for an urgent and concerted hypertension strategy to reduce a mounting cardiovascular diseases burden. This would be critical to prevent or delay target organ damage such as stroke and chronic kidney disease which are especially common among blacks with a tendency to affect the most productive base of the population.^18^, ^19^, ^20^ Additionally, these complications will put further strain on the country's meagre health resources.^21^

The findings in our study are consistent with reports in other low-income countries.^5^^,^^22^^,^^23^ High-income countries such as the United States and Canada have greatly improved hypertension prevention, detection and management through effective population strategies, improved healthcare access and blood pressure measurements.^24^ The Gambia like others in the sub-region, have considerable challenges in implementing such comprehensive programmes. The health system continues to be plagued by inadequate and inequitable distribution of physicians, support services personnel, infrastructure and equipment.^25^ There should therefore be a more consistent supply and maintenance of basic equipment such as blood pressure monitors for diagnosis and management of patients. Community-based programmes to improve hypertension control and increase awareness and education about hypertension and its risk factors, as being implemented in SSA,^26^^,^^27^ may greatly improve outcomes. There is also a critical shortage of physicians to care for the large number of patients with the national health workforce index currently at 1.53 per 1000 population against the WHO's recommended 4.45 per 1000 population.^28^ Our results do not show any difference at any stage of the care cascade by rural-urban location. In The Gambia where majority of population reside in urban areas, lifestyles are increasingly similar regardless of location. Travel between areas is common especially during the dry season when farming activities are at a minimum.

The prevalence of hypertension recorded in this study is considerably higher than previously reported in The Gambia. Though there have only been a few nationally representative studies in The Gambia, the estimates are not directly comparable given the difference in age of participants included in the respective studies and the age standardisation in our study. The nationwide survey in 1997 including participants aged ≥15 years found a prevalence of 24.1%^29^ whilst the prevalence in the 2010 STEPS survey including adults aged 25–64 years was 29%.^12^ However, the findings are consistent with those of Akpa et al. in native Africans with a mean age of 48.5 years from 13 countries.^30^

Overall, we observed a better cascade of care performance in women compared to men. This could be attributed to the greater healthcare utilization by women compared to men as has been widely reported.^31^, ^32^, ^33^, ^34^, ^35^ Compared to studies other low income settings such as in Senegal,^36^ and Sierra Leone,^37^ the overall control rates was similar in men and women, although women were more likely to be aware of their status. However, we observed that women in the older age categories (70 years and above) were less likely to be retained in the care process. This age group may be more vulnerable with possibly greater financial and other barriers to accessing healthcare.^38^ Though our study found that women were more likely to be treated for hypertension, there was however no significant difference in the odds of achieving BP control. This is consistent with reports elsewhere^39^^,^^40^ suggesting the need to explore a sex-specific approach for hypertension treatment owing to differences in presentation and progression.

The biggest loss in the hypertension care cascade in our study was from the stage of being hypertensive to being diagnosed. This was nearly half of all hypertensive patients, noting a higher loss in men (56.4%) than in women (35.2%). The proportion of undiagnosed hypertension was significantly higher in the 2010 STEPS survey (79% overall; 86% in men and 71.4% in women). This, as in our study, found younger populations to be disproportionately affected.^12^ Perceptions and health-seeking behaviours of young people, especially as it differs from those of older generations, should be explored for more targeted intervention in sub-Saharan Africa. Johnson and colleagues, in exploring barriers to hypertension care in the United States found that young people did not usually take a hypertension diagnosis well and were surprised and angry about a hypertension diagnosis.^41^ They generally expected to develop hypertension at a much older age and perceived that a hypertension diagnosis negatively altered their young identity.^41^

Our study as in the 2010 STEPS survey^12^ found obese individuals to be more likely to have their hypertension status detected. This is not surprising given their greater contact with healthcare, due to the presence of comorbidities, and therefore they are more likely to be screened and diagnosed.^42^^,^^43^

We observed that the treatment coverage for hypertension i.e., diagnosed patients who were receiving treatment was relatively high at 70.4%. However, barriers to treatment in the remaining patients should be investigated and addressed. Socio-economic challenges could be a huge factor as medication for hypertension require usage in the long term and are generally beyond reach to many financially disadvantaged populations.^44^ There is still limited knowledge or misunderstanding on hypertension in both patients and healthcare providers. A lot of patients fear initiating antihypertensive treatment and commonly resort to traditional practices even after being initiated on treatment, as has been reported in a study in 12 African countries.^45^ The limited understanding and the asymptomatic nature of the condition also has a big impact on medication adherence.^46^^,^^47^

We found that only 10% of hypertensive patients and 24.2% of those receiving treatment had a controlled blood pressure. These respective rates in our study were higher than those observed in the sub-region. Macia et al. in older Senegalese adults of ≥50 years found 6.7% of hypertensives and 17.4% of treated patients to have a controlled blood pressure.^36^ Geraedts et al. found these to be 5% and 11% respectively in Sierra Leoneans aged ≥18 years.^37^ A meta-analysis of 33 surveys in sub-Saharan Africa with a mean age of 40 years found only 7% were retained in the hypertension care cascade.^48^ Beyond offering treatment to patients, other factors limiting treatment success should be investigated. Commonly used guidelines for treating patients in sub-Saharan Africa are mostly extrapolations from data derived from the diaspora Africans in the United States. This requires caution as differences in socio-economic status, cardiovascular risk and response to antihypertensive drug treatment exist.^49^ Other potential factors affecting treatment success could be lack of sufficient and constant supply of high-quality drugs, and where available, the level of adherence by patients. The latter could be improved by reducing pill burden through the provision of combination therapy.^50^^,^^51^

Our study has several strengths including the use of a nationally representative sample. However, the results should be considered in light of some limitations. We only included adults aged 35 years and above, so results are not generalisable to younger age groups. Diagnosis of hypertension also included an element of self-report as part of the composite definition of hypertension which may have introduced bias. Our inclusion of potential confounders in multivariate analysis may not be exhaustive and we cannot therefore rule out the possibility of residual confounding. Furthermore, we used only a cross-sectional measurement of blood pressure when current clinical approaches require several measurements at different timepoints. Our assessment also only considered drug treatment and did not include other lifestyle approaches. Furthermore, our assessment of treatment status for hypertension was based on participant report, and hence the possibility of recall bias could not be ruled out.

In conclusion, the data shows that improvements are required at all stages of the cascade. The greatest dividends will be gained in reducing the mounting prevalence and improving diagnosis of patients with hypertension, the stage where the greatest loss in the cascade occur. Currently, there are insufficient population approaches for hypertension prevention. The high prevalence of hypertension should therefore be addressed through a comprehensive national multisectoral strategy to increase public awareness about hypertension, as well as providing information on preventative methods including dietary and lifestyle modifications. Policies against the selling and consumption of sodium-rich, and energy dense foods respectively should be formulated and implemented. There should also be increased population screening for hypertension using a community-centred hypertension programme. Blood pressure monitors should be provided to health facilities to ease mass screening of patients and otherwise healthy population wishing to have their blood pressure measured. This will require training and recruitment of more healthcare personnel, as well as adopting task-shifting approaches to increase access to diagnosis and management of hypertension. There should also further research, besides greater efforts to improve adherence to understand reasons for low control rates among treated patients. Such research should systematically assess the health service and system structures, strengthen the evidence base for how to identify those who would benefit most from treatment and to find better approaches for risk stratification that will work in a low-income setting.

## Contributors

MJB acquired the funding for this study. MJ, IM, AH, AMP and MJB conceived the study. MJ, IM and MJK curated and validated the data. MJ, IM, SB, AH, AMP, and MJB designed and implemented the study. OB supported the implementation of the study. MJ conducted the literature review, with support from AMP and PP. MJ and MJK performed the analysis. AMP, MJB, IM, OB, MB and PP advised on analysis and interpretation of the data. MJ drafted the manuscript. IM, SB, MJK, AH, MB, OB, PP, AMP and MJB revised the manuscript. All authors had final responsibility for the decision to submit for publication.

## Data sharing statement

Survey content is available upon request. For any data requests, please contact Islay Mactaggart (Islay.Mactaggart@lshtm.ac.uk).

## Declaration of interests

None.

