# Supplementary material for: Evaluating the hypertension care cascade in middle-aged and older adults in The Gambia: findings from a nationwide survey
Source: eClinicalMedicine. 2023 Sep 20;64:102226. doi: 10.1016/j.eclinm.2023.102226 (PMC10520336; doi:10.1016/j.eclinm.2023.102226)
Supplement: Supplementary material [file mmc1.docx]

**Evaluating the hypertension care cascade in middle-aged and older adults in The Gambia: Findings from a nationwide survey**

**Short title: Hypertension care cascade in Gambian adults**

**Authors:** Modou Jobe^1,*^, Islay Mactaggart^2^, Abba Hydara^3^, Min J Kim^2^, Suzannah Bell^4^, Omar Badjie^5^, Mustapha Bittaye^6,7^, Andrew M Prentice^1^, Matthew Burton^2,8^

**Supplementary appendix**

Supplementary Table 1: Age and sex-standardised prevalence, diagnosis, treatment, and control of hypertension by age and sex

|  |  |  |  |  |  | **Controlled** | |
| --- | --- | --- | --- | --- | --- | --- | --- |
| **Variable** | **Number of participants** | **Number of individuals with hypertension** | **Prevalence of hypertension** | **Proportion with diagnosed hypertension (aware)** | **Proportion of hypertensive patients treated** | **Among all hypertensives** | **Among treated** |
| **Men** |  |  |  |  |  |  |  |
| 35-44 | 1952 | 821 | 28.5 (25.3-31.7) | 23.8 (17.6-30.1) | 60.4 (46.4-74.4) | 6.7 (3.1-10.2) | 41.2 (24.2-58.3) |
| 45-54 | 1259 | 419 | 43.2 (38.3-47.5) | 47.3 (40.8-53.8) | 65.7 (55.9-75.5) | 6.7 (3.7-9.7) | 21.6 (12.1-31.1) |
| 55-64 | 715 | 149 | 64.6 (60.6-68.6) | 50.2 (44.8-55.6) | 71.9 (65.4-78.5) | 6.6 (4.0-9.2) | 17.5 (11.1-23.9) |
| 65-74 | 414 | 66 | 73.0 (69.0-77.0) | 55.1 (49.9-60.3) | 75.2 (69.0-82.2) | 6.5 (3.9-9.1) | 15.3 (9.5-21.2) |
| 75-84 | 184 | 27 | 74.9 (68.4-81.4) | 58.0 (49.5-66.6) | 73.6 (62.7-84.5) | 8.2 (3.4-13.0) | 18.3 (8.1-28.5) |
| 85+ | 74 | 12 | 73.2 (62.1-84.3) | 53.7 (37.3-70.1) | 85.1 (66.4-1.03) | 8.2 (0.3-16.1) | 21.9 (2.7-16.8) |
| All | 4598 | 1494 | 44.7 (42.4-47.0) | 43.6 (40.5-46.8) | 69.5 (65.1-73.9) | 6.8 (5.3-8.2) | 21.7 (17.5-25.9) |
|  |  |  |  |  |  |  |  |
| **Women** |  |  |  |  |  |  |  |
| 35-44 | 2043 | 328 | 32.4 (30.6-34.2) | 57.3 (53.8-60.8) | 61.1 (56.5-65.6) | 15.1 (12.5-17.7) | 35.6 (30.5-40.7) |
| 45-54 | 1203 | 319 | 52.8 (50.0-55.6) | 64.1 (60.4-67.7) | 71.8 (67.5-76.2) | 14.5 (11.9-17.0) | 28.0 (23.5-32.6) |
| 55-64 | 635 | 271 | 65.0 (61.9-68.1) | 69.5 (65.9-73.1) | 75.4 (70.6-80.2) | 12.9 (10.1-15.7) | 23.2 (18.4-28.0) |
| 65-74 | 393 | 178 | 76.8 (72.8-80.8) | 72.6 (68.1-77.0) | 75.2 (69.5-81.0) | 10.6 (7.7-13.6) | 19.0 (14.0-24.1) |
| 75-84 | 209 | 81 | 79.4 (74.4-84.4) | 70.2 (63.0-77.4) | 86.4 (80.3-92.5) | 6.7 (3.3-10.1) | 11.2 (5.5-16.8) |
| 85+ | 106 | 32 | 82.1 (72.0-92.2) | 68.4 (54.1-82.6) | 72.6 (56.9-88.2) | 6.5 (-0.6-13.7) | 12.8 (-1.0-26.5) |
| All | 4589 | 1209 | 49.3 (47.8-50.8) | 64.8 (62.7-66.9) | 71.0 (67.9-74.0) | 13.0 (11.7-14.3) | 25.6 (23.3-27.9) |

Supplementary Table 2: Age and sex-standardised prevalence, diagnosis, treatment, and control of hypertension by sex and location

|  | **Overall** | | **Urban** | | **Rural** | |
| --- | --- | --- | --- | --- | --- | --- |
|  | **Men** | **Women** | **Men** | **Women** | **Men** | **Women** |
| **Prevalence** | 44.7 (42.4-47.0) | 49.3 (47.8-50.8) | 43.5 (40.4-46.5) | 49.0 (47.2-50.9) | 46.0 (42.6-49.3) | 49.7 (47.2-52.2) |
| **Diagnosed** | 43.6 (40.5-46.8) | 64.8 (62.7-66.9) | 43.3 (38.8-47.8) | 63.6 (60.9-66.4) | 43.9 (39.5-48.4) | 66.5 (63.2-69.7) |
| **Treated** | 73.8 (69.4-78.2) | 79.4 (76.6-82.3) | 71.1 (64.9-77.3) | 77.7 (73.6-81.8) | 76.4 (70.1-82.7) | 81.7 (77.9-85.6) |
| **Controlled (among hypertensives)** | 6.8 (5.3-8.2) | 13.0 (11.7-14.3) | 5.6 (3.9-7.4) | 12.5 (10.7-14.2) | 7.8 (5.6-10.1) | 13.7 (11.7-15.7) |
| **Controlled (among treated)** | 21.7 (17.5-25.9) | 25.6 (23.3-27.9) | 18.5 (13.0-24.0) | 25.4 (22.3-28.5) | 24.6 (18.4-30.8) | 25.8 (22.4-29.2) |
